# Comparative outcomes of conservative, steinmann pin, and plate fixation in calcaneal fractures: a subtype-based evaluation according to the essex-lopresti classification

**DOI:** 10.1186/s13018-025-06533-1

**Published:** 2025-12-02

**Authors:** Ömer Esmez, Şükrü Demir, Murat Gürger, Arif Gülkesen, Furkan Bilek

**Affiliations:** 1 Fethi Sekin City Hospital, Department of Orthopedics, 23100 Elazığ, Turkey; 2 Faculty of Medicine, Department of Orthopedics, Fırat University, 23119 Elazığ, Turkey; 3 Faculty of Medicine, Department of Physical Medicine and Rehabilitation , Fırat University, 23119 Elazığ, Turkey; 4 Fethiye Faculty of Health Sciences, Department of Gerontology, Muğla Sıtkı Koçman University, 48330 Muğla, Turkey

**Keywords:** Calcaneal fracture, Essex-Lopresti classification, Steinmann pin, Plate fixation, Gait analysis

## Abstract

**Background:**

Calcaneal fractures are the most common tarsal fractures and often result in long-term disability. Although various treatment options exist, but the relationship between Essex-Lopresti subtypes, treatment methods, and dynamic functional recovery remains unclear.

**Methods:**

This retrospective study included 66 patients with intra-articular calcaneal fractures, treated between 2011 and 2021. Fractures were categorized according to the Essex-Lopresti classification (1 A–1 C, 2 A–2 C) and managed by conservative treatment, Steinmann pin fixation, or plate fixation. Functional outcomes were assessed, using the American Orthopaedic Foot and Ankle Society (AOFAS) score, radiographic parameters (Böhler and Gissane angles), and pedographic gait analysis with the Win-Track platform. Statistical analysis was performed using Kruskal–Wallis tests with Dunn–Bonferroni post-hoc analyses, Mann–Whitney U or independent-samples t-tests as appropriate, chi-square (or Fisher’s exact) tests for categorical variables, and Spearman’s rho for correlation.

**Results:**

Functional outcomes varied across subtypes and treatment methods. Across subtypes, the distribution of AOFAS categories did not differ significantly (χ², *p* = 0.587). Type 2 A fractures treated with Steinmann pin fixation demonstrated the highest AOFAS scores (80.4 ± 10.2; *p* = 0.587). Böhler’s angle was numerically higher in the conservative group (17.0 ± 11.4°) but did not correlate with AOFAS scores (ρ = 0.01, *p* = 0.94). Pedographic analysis showed that maximum plantar pressure was highest in the conservative group (1625 ± 142 kPa) and lowest in the plate fixation group (1437 ± 188 kPa; overall *p* = 0.033). Gait asymmetries—particularly prolonged swing and stride duration tended to be greater in the Type 2B and 2 C subgroups, although statistical significance was limited ((*p* = 0.195–0.795)).

**Conclusion:**

Essex-Lopresti subtypes strongly influence clinical and gait outcomes following calcaneal fractures. Steinmann pin fixation is advantageous in Type 2 A fractures, while Type 2B fractures consistently show poor recovery. Radiographic angles alone are insufficient predictors of long-term outcomes, emphasizing the importance of integrating gait analysis with clinical scoring. Subtype-specific approaches may optimize treatment strategies and patient care.

**IRB number:**

Ethics Committee of Fırat University (2022/04–04).

**Supplementary Information:**

The online version contains supplementary material available at 10.1186/s13018-025-06533-1.

## Background

 Calcaneal fractures represent approximately 1–2% of all fractures in the human body, with an incidence of 11.5 per 100,000 per year [1–3]. Among the tarsal bones, they are the most common and typically occur after high-energy trauma such as a fall from height. Concomitant injuries are frequent, including vertebral fractures (10–20%), contralateral calcaneus fractures (7–10%), and various extremity injuries (26%) [4, 5]. The intra-articular joint defects that arise from these fractures often result in chronic pain, functional limitations, and long-term disability [4, 5].

Treatment strategies for calcaneal fractures include conservative management, closed reduction with Kirschner wires, Steinmann pinning, and open reduction with internal fixation (ORIF) [6]. The primary objective is to achieve early functional recovery by restoring anatomical alignment [7]. Although conservative treatment allows for an earlier return to daily activities, ORIF generally provides superior functional outcomes owing to improved restoration of calcaneal height, width, and heel morphology [8, 9].

Despite these surgical advantages, calcaneal fractures often lead to significant alterations in gait mechanics and load distribution. Postoperative gait analyses frequently demonstrate persistent irregularities despite radiological evidence of adequate reduction [10, 11]. Plantar pressure studies have shown that plate fixation in unilateral intra-articular fractures may restore plantar load distribution to a degree comparable with the uninjured side, correlating with improved functional outcomes [11, 12]. Rosenbaum et al. also reported pedobarographic similarities between conservative and surgical treatment, although the sample size was limited [13].

Radiological measures such as Böhler and Gissane angles have traditionally been used to assess reduction quality, yet their correlation with long-term functional outcomesremains inconsistent [14–16]. More recently, pedographic assessment and spatiotemporal gait analysis have emerged as complementary tools to evaluate dynamic recovery [17]. The Essex–Lopresti classification, which categorizes intra-articular calcaneal fractures according to the direction of the primary fracture line and the displacement pattern of the posterior facet, plays an essential role in predicting surgical difficulty and long-term functional recovery. Nevertheless, few studies have correlated these subtypes with objective gait parameters that reflect real-world biomechanical function. Therefore, this study integrates radiological classification with dynamic pedographic assessment to clarify how morphological differences translate into functional performance [18]. Calcaneal fractures remain a challenging injury due to their complex anatomy and high rate of postoperative complications. Recent studies have highlighted that complications such as wound healing problems, subtalar joint stiffness, and chronic pain remain common following both operative and non-operative management of calcaneal fractures [19–21]. The risk of soft-tissue complications, including infection and skin necrosis, is particularly notable in patients treated with extensile lateral approaches, underscoring the importance of less invasive fixation techniques and careful patient selection [19–21]. A previous review emphasized the ongoing debate regarding the optimal management of calcaneal fractures. The systematic evaluation of randomized trials indicated that although surgical fixation can improve anatomical restoration, there is limited evidence supporting its clear long-term functional superiority over conservative treatment [22]. More recent research demonstrated that displaced intra-articular calcaneal fractures are frequently associated with considerable functional impairment and wound-related complications, even following operative management [23]. These findings frame the rationale for our subtype-based comparison of conservative, Steinmann pin, and plate fixation outcomes.

The present study aims to address this gap by comparing conservative, Steinmann pin, and plate fixation techniques in calcaneal fractures stratified by Essex-Lopresti subtypes (1 A–1 C and 2 A–2 C). Clinical outcomes were assessed using the American Orthopaedic Foot and Ankle Society (AOFAS) scoring system, while dynamic recovery was evaluated through pedographic and gait analysis [24]. We hypothesize that minimally invasive approaches may achieve functional results comparable to plate fixation when stratified by fracture subtype. To the best of our knowledge, this is one of the first studies to specifically correlate Essex-Lopresti subtypes with both clinical outcomes and gait analysis parameters, thereby providing novel insights into optimizing treatment strategies.

## Methods

### Study design and participants

This retrospective and cross-sectional study was conducted at a tertiary university hospital between November 2011 and November 2021. Patients diagnosed with calcaneal fractures by an orthopedic specialist were evaluated, with or without surgical intervention. Initially, 103 patients were screened, and those meeting the following exclusion criteria were omitted: (1) fracture sustained < 18 months prior to evaluation, (2) bilateral fractures, (3) isolated non-calcaneal fractures (e.g., talus, navicular, or metatarsals), (4) age < 18 years, (5) ipsilateral lower extremity loss, and (6) inability to contact or refusal to participate.

After exclusions, 66 patients (58 males, 8 females) were included in the final analysis. The 18-month threshold was set to ensure sufficient time for fracture healing and gait adaptation, although full recovery may extend beyond this period.

Patients were divided into three groups according to the treatment method:

- Group 1 (*n* = 12): Conservative treatment with plaster immobilization.
- Group 2 (*n* = 42): Closed reduction and percutaneous fixation using Steinmann pins.
- Group 3 (*n* = 12): Open reduction and internal fixation with a plate.

No statistically significant differences were found among the groups regarding demographic characteristics (Table 1). Treatment protocols adhered to institutional guidelines (detailed in Supplementary File S1), including cast immobilization for 6–8 weeks, staged weight-bearing, and postoperative rehabilitation tailored to fracture severity and surgical approach.

**Table 1:** Demographic data according to Essex-Lopresti subtypes

Subtype	*n*	Age (years, mean ± SD)	Sex (male/female)	Height (cm, mean ± SD)	BMI (mean ± SD)	Side (*R*/L)	Smoking (yes/no)	Diabetes (yes/no)	Mechanism of injury (fall/traffic/other)	Occupational status (heavy labor/clerk/other)
Type 1 A	7	41.2 ± 10.5	6/1	166.6 ± 7.2	27.9 ± 3.1	4/3	3/4	1/6	6/1/0	4/2/1
Type 1B	14	42.8 ± 9.3	12/2	175.6 ± 6.5	26.1 ± 2.8	7/7	5/9	2/12	11/2/1	8/3/3
Type 1 C	3	40.1 ± 11.2	3/0	173.3 ± 5.4	22.3 ± 2.5	2/1	1/2	0/3	2/1/0	1/1/1
Type 2 A	11	43.5 ± 10.8	10/1	170.9 ± 6.3	25.6 ± 3.2	5/6	4/7	3/8	8/2/1	6/3/2
Type 2B	12	45.2 ± 9.9	11/1	169.2 ± 7.5	24.8 ± 2.7	6/6	7/5	2/10	10/1/1	7/4/1
Type 2 C	19	44.0 ± 8.7	16/3	170.1 ± 6.9	25.9 ± 3.0	10/9	9/10	4/15	15/3/1	9/6/4

**Table 2:** AOFAS scores according to Essex-Lopresti subtypes

Subtype	*n*	AOFAS (mean ± SD)	Excellent (≥ 90)	Good (80–89)	Fair (70–79)	Poor (< 70)
Type 1 A	7	76.3 ± 12.8	0 (0.0%)	4 (57.1%)	2 (28.6%)	1 (14.3%)
Type 1B	14	78.5 ± 18.8	6 (42.9%)	3 (21.4%)	1 (7.1%)	4 (28.6%)
Type 1 C	3	72.0 ± 16.7	1 (33.3%)	0 (0.0%)	0 (0.0%)	2 (66.7%)
Type 2 A	11	80.4 ± 10.2	2 (18.2%)	5 (45.5%)	2 (18.2%)	2 (18.2%)
Type 2B	12	69.7 ± 19.2	2 (16.7%)	3 (25.0%)	2 (16.7%)	5 (41.7%)
Type 2 C	19	78.5 ± 17.4	4 (23.5%)	6 (35.3%)	2 (11.8%)	5 (29.4%)
Overall	*p* = 0.587					

**Table 3:** Radiological parameters (Böhler and Gissane Angles)

Parameter	Conservative (mean ± SD)	Steinmann pin (mean ± SD)	Plate fixation (mean ± SD)	Overall (*p*)	Conservative–steinmann (*p*)	Steinmann–plate (*p*)	Plate–conservative (*p*)
Böhler Angle (°)	17.0 ± 11.4	15.9 ± 12.1	13.1 ± 9.7	0.726	0.812	0.543	0.392
Gissane Angle (°)	114.8 ± 13.8	114.8 ± 13.7	114.4 ± 13.8	0.979	0.996	0.944	0.951

**Table 4:** Pedographic and spatiotemporal parameters – fractured side

Parameter (mean ± SD)	Conservative	Steinmann	Plate	Overall *p*	Conservative–steinmann (*p*)	Steinmann–plate (*p*)	Plate–conservative (*p*)
Maximum plantar pressure (kPa)	1625 ± 142	1506 ± 283	1437 ± 188	0.033*	0.037*	0.371	0.010*
Swing duration (ms)	1327 ± 155	1495 ± 245	1525 ± 276	0.031*	0.009*	0.942	0.069
Stride duration (ms)	1971 ± 214	2100 ± 262	2103 ± 246	0.713	0.407	0.933	0.639
Foot angle (°)	8 ± 7	8 ± 3	6 ± 4	0.362	0.399	0.208	0.583
Step duration (ms)	582 ± 92	643 ± 114	657 ± 139	0.080	0.023*	0.983	0.138
Gait cycle duration (ms)	1132 ± 214	1317 ± 236	1304 ± 212	0.219	0.090	0.913	0.208
Double stance duration (ms)	397 ± 103	397 ± 109	401 ± 83	0.913	0.721	0.817	0.792
Single stance duration (ms)	418 ± 89	435 ± 97	440 ± 85	0.622	0.411	0.734	0.568
Swing duration (non-fractured side, ms)	1361 ± 200	1512 ± 353	1610 ± 355	0.082	0.062	0.328	0.060
Stride duration (non-fractured side, ms)	2039 ± 227	2069 ± 318	2241 ± 334	0.079	0.198	0.095	0.054
Step length (cm)	49 ± 12	49 ± 7	50 ± 9	0.653	0.453	0.505	0.954
Cadence (steps/min)	103.0 ± 17.4	96.0 ± 19.0	99.4 ± 36.9	0.346	0.194	0.678	0.229

**Table 5:** Comparative gait parameters by Essex-Lopresti subtypes - fractured side

Parameter (mean ± SD)	Type 1 A	Type 1B	Type 1 C	Type 2 A	Type 2B	Type 2 C	*p*-value
Maximum plantar pressure (kPa)	1518.1 ± 300.5	1606.6 ± 209.2	1405.7 ± 81.8	1506.1 ± 163.8	1466.8 ± 357.6	1592.9 ± 251.9	0.284
Swing duration (ms)	1517.1 ± 237.6	1422.9 ± 145.0	1570.0 ± 217.8	1472.5 ± 241.5	1497.1 ± 408.6	1427.6 ± 174.0	0.795
Stride duration (ms)	2133.5 ± 166.5	2042.2 ± 202.8	2178.8 ± 139.5	2074.7 ± 188.3	2056.2 ± 208.2	2060.4 ± 150.8	0.664
Foot angle (°)	7.5 ± 2.1	9.0 ± 4.1	6.9 ± 4.3	6.6 ± 2.1	8.6 ± 3.5	6.7 ± 3.0	0.372
Step duration (ms)	659.3 ± 111.9	629.6 ± 70.8	690.0 ± 98.4	661.8 ± 202.8	740.4 ± 465.1	619.1 ± 93.7	0.761
Gait cycle duration (ms)	1312.9 ± 199.1	1244.6 ± 99.0	1320.0 ± 128.2	1348.2 ± 395.0	1269.2 ± 203.7	1268.5 ± 125.1	0.927
Double stance duration (ms)	409.0 ± 55.6	459.9 ± 183.5	435.3 ± 68.7	392.4 ± 53.2	372.3 ± 44.3	401.4 ± 44.4	0.298
Single stance duration (ms)	430.0 ± 55.5	440.0 ± 56.6	300.0 ± 13.3	396.7 ± 15.3	405.0 ± 64.5	540.0 ± 99.0	0.195
Swing duration (non-fractured side, ms)	1420.5 ± 210.3	1389.6 ± 194.2	1458.2 ± 201.5	1501.3 ± 220.4	1489.1 ± 211.6	1524.7 ± 229.5	0.728
Stride duration (non-fractured side, ms)	2078.5 ± 195.1	2050.8 ± 211.9	2105.0 ± 223.4	2089.2 ± 229.8	2092.0 ± 232.2	2135.6 ± 248.4	0.701
Step length (cm)	51.44 ± 5.36	52.69 ± 11.01	46.08 ± 8.13	48.25 ± 5.68	48.85 ± 7.68	52.16 ± 6.80	0.203
Cadence (steps/min)	94.4 ± 17.5	92.2 ± 13.4	82.5 ± 13.9	93.9 ± 21.3	103.0 ± 21.6	107.1 ± 29.9	0.186

Treatment allocation was not randomized. The attending orthopedic surgeon determined the appropriate method based on fracture morphology, soft tissue condition, comorbidities (e.g., diabetes mellitus, smoking), and patient-specific functional demands. The absence of a standardized allocation system (e.g., Sanders-based) was acknowledged as a study limitation and discussed accordingly.

### Data collection tools

#### Spatiotemporal parameters

Dynamic plantar pressure and gait parameters were evaluated using the Win-Track gait platform (MEDICAPTEURS Technology, France). The platform dimensions were 1610 × 652 × 30 mm, with a sensor thickness of 9 mm. All gait assessments were performed by an experienced physiotherapist who was blinded to both the treatment method and fracture subtype. The assessor used anonymized participant codes during data collection and analysis to minimize measurement bias. The system was calibrated according to the manufacturer’s protocol before each session.

**Fig. 1:**
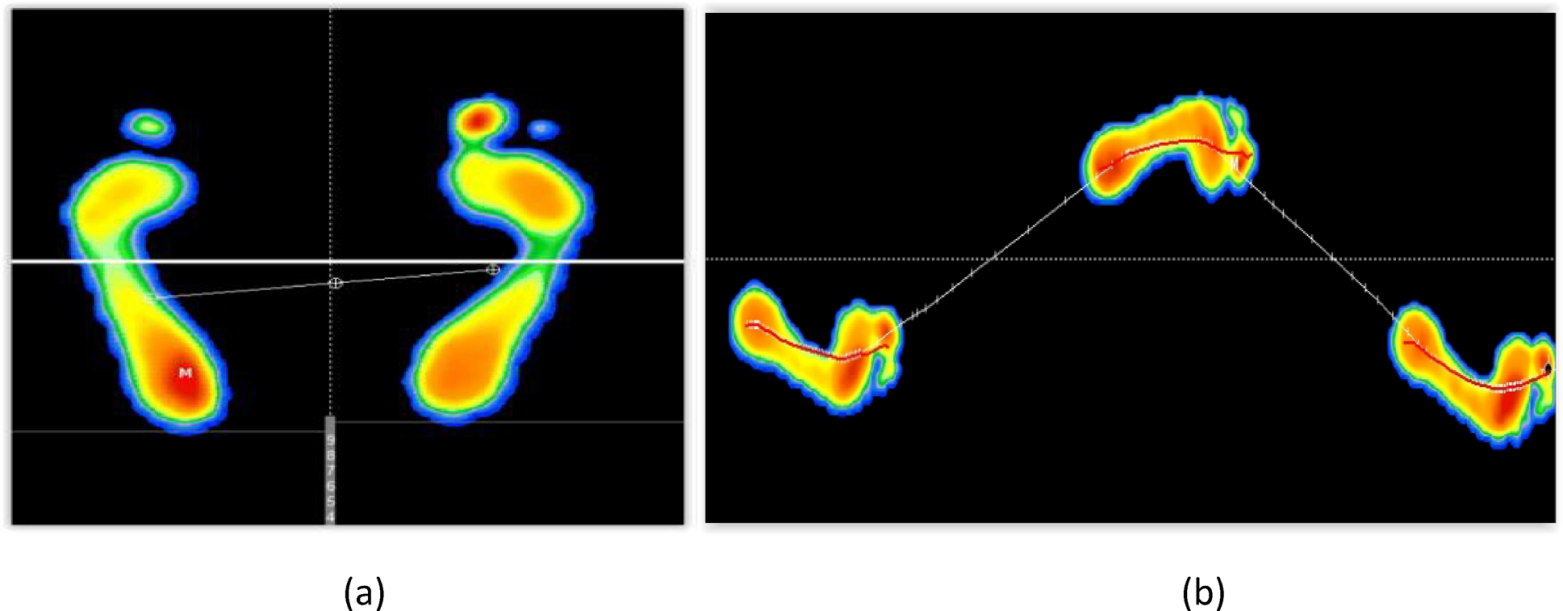
A detailed examination of the spatiotemporal parameters of gait as captured by the Win-Track platform. The images on the left depict a static posture (**a**), while those on the right illustrate a dynamic gait (**b**). The images represent an anonymized participant with a Type 2 A calcaneal fracture treated with Steinmann pin fixation. All gait data and images were obtained directly from study participants

Patients were instructed to walk barefoot at a self-selected comfortable speed, and at least three valid gait cycles were recorded for each individual. Each trial included one full gait cycle for both limbs, and the mean values of all trials were used to minimize inter-trial variability. The system recorded both static postural and dynamic spatiotemporal parameters, including step duration (ms), step length (cm), stride duration (ms), cadence (steps/min), double-support time (ms), foot progression angle (°), and maximum plantar pressure (kPa). Data were automatically processed by Win-Track software (version 7.1) and exported for statistical analysis [25] (Figs. 1).

#### Radiological evaluation

To evaluate the relationship between fracture morphology and dynamic performance, gait data were analyzed according to the Essex-Lopresti classification, which divides intra-articular calcaneal fractures into six subtypes (1 A–1 C and 2 A–2 C) based on the direction of the primary fracture line and the displacement of the posterior facet [18]. This approach enabled correlation of radiological subtype characteristics with pedobarographic outcomes, providing an objective indicator of real-world biomechanical recovery.

**Fig. 2:**
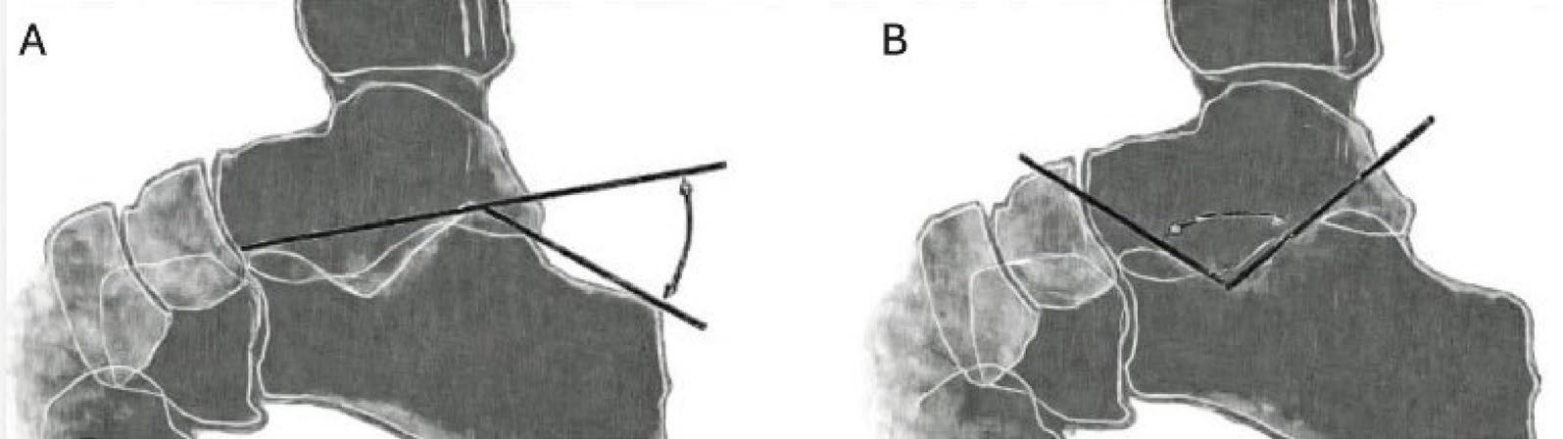
Schematic representations of Böhler’s (**a**) and Gissane’s (**b**) angles on lateral views of calcaneal fractures

Radiological evaluation included measurement of Böhler’s and Gissane’s angles on lateral radiographs (Fig. 2). Böhler’s angle is defined by lines connecting the anterior process, posterior articular facet, and calcaneal tuberosity, normally ranging between 20°–40°; lower values indicate posterior facet depression. Gissane’s angle, formed between the posterior facet and anterior process, normally measures 120°–145° and typically increases in displaced fractures.

All measurements were performed by two orthopedic surgeons using Picture Archiving and Communication System digital tools, demonstrating excellent interobserver reliability (intraclass correlation coefficient = 0.91) [15, 18].

#### American orthopaedic foot and ankle society

Functional outcomes were evaluated using the AOFAS Ankle-Hindfoot core. This scale consists of three subdomains: pain (40 points), function (50 points), and alignment (10 points). A total score ≥ 90 was considered excellent, 80–89 good, 70–79 fair, and < 70 poor. The Turkish version of the AOFAS scale has previously been validated for reliability and internal consistency [24].

### Ethical approval

The study protocol was approved by the Non-Interventional Research Ethics Committee of Fırat University (Session No: 2022/04-04) and conducted in accordance with the principles of the Declaration of Helsinki. Written informed consent was obtained from all participants prior to inclusion.

### Statistical analysis

The data were digitized using the Statistical Package for the Social Sciences (SPSS), version 22.0 (SPSS Inc., Chicago, IL, USA). Continuous variables were presented as mean ± standard deviation (SD). The normality of the AOFAS scores, range of motion measurements, and radiological data was assessed using the Shapiro-Wilk test. As the data did not follow a normal distribution, non-parametric tests were employed for statistical analyses. Group comparisons were performed using the Kruskal-Wallis test for multiple groups and the Mann-Whitney U test for pairwise comparisons. Categorical variables, including gender distribution and AOFAS grade differences between groups, were analyzed using the chi-square test. A* p*-value of <0.05 was considered statistically significant. For multiple group comparisons, the Kruskal–Wallis test was used, followed by Dunn–Bonferroni post-hoc analyses when overall significance was observed. No post-hoc tests were applied for non-significant omnibus results (e.g., Table 5). We performed a post-hoc power analysis using G*Power 3.1, which revealed that our sample size achieved 80% power to detect large effect sizes (d=0.8) in gait parameters between groups at α=0.05. However, we recognize the reduced sensitivity to detect smaller, potentially clinically meaningful differences, particularly between the smaller conservative and plate fixation groups.

## Results

### Demographic findings 

A total of 66 patients were included and stratified into Essex-Lopresti subgroups: Type 1A (n=7), Type 1B (n=14), Type 1C (n=3), Type 2A (n=11), Type 2B (n=12), and Type 2C (n=19). No statistically significant differences were found among fracture subtypes in terms of age, sex, BMI, smoking status, diabetes, or side of injury (p = 0.20 – 0.95; Table 1).

### Functional outcomes (AOFAS Score)

The mean AOFAS scores varied across the fracture subtypes. Type 1 subtypes generally demonstrated balanced results, although the Type 1C subgroup showed relatively lower scores. In Type 2A fractures, patients treated with Steinmann pin fixation achieved the highest scores (mean 80.4 ± 10.2; p = 0.587). Among treatment groups, Steinmann pin fixation in Type 2A fractures yielded the highest proportion of favorable results, with 18.2% of patients achieving excellent and 45.5% achieving good AOFAS scores (Table 2). By contrast, Type 2B fractures showed a higher proportion of poor outcomes (5/12, 41.7% vs 2/11, 18.2%), but this difference was not statistically significant (Fisher’s exact test, p = 0.37). Conversely, Type 2B fractures were associated with significantly lower functional outcomes (mean 69.7 ± 19.2; p = 0.37). In Type 2C fractures, patients treated with Steinmann pin fixation obtained results comparable to those managed with plate fixation (p = 0.587). These findings indicate that functional success differed according to both the Essex-Lopresti subtype and the treatment method (Table 2). 

### Radiological findings

When Böhler’s and Gissane’s angles were evaluated across treatment groups, no significant differences were detected (Table 3). Böhler’s angle was numerically higher in the conservative group (17.0 ± 11.4°) and lowest in the plate fixation group (13.1 ± 9.7°), but these differences were not statistically significant (Kruskal–Wallis, p = 0.726). Gissane’s angle also did not differ significantly among groups (p = 0.979). These findings suggest that radiographic correction does not necessarily correlate with functional outcomes. (Table 3). 

### Gait analysis findings 

Pedographic and spatiotemporal gait analysis using the Win-Track platform demonstrated that maximum plantar pressure was highest in the conservative group and lowest in the plate fixation group (p = 0.033; Table 4). Step duration was significantly longer in the Steinmann group than in the conservative group (p = 0.023), while comparisons involving the plate group were not significant (all p ≥ 0.138; Table 4).In the Essex–Lopresti subtype analysis, swing duration tended to be longer in Type 2B and 2C fractures, reflecting persistent gait imbalance, whereas other spatiotemporal parameters—including stride duration, double-stance time, and foot angle—did not differ significantly among subtypes (p = 0.795; Table 5).These findings indicate that Steinmann pin fixation provided the most favorable gait performance in Type 2A fractures, while Type 2B fractures exhibited persistent functional impairment and irregular load distribution regardless of treatment. In Type 2C fractures, functional gait outcomes were largely comparable between Steinmann pin and plate fixation (p = 0.284 - 0.795; Table 5).

## Discussion 

This study provides a comparative evaluation of conservative management, Steinmann pin fixation, and plate fixation in calcaneal fractures stratified by Essex-Lopresti subtypes, focusing on their clinical, radiographic, and dynamic gait outcomes. To our knowledge, this is one of the few studies that integrates pedographic gait analysis with subtype-specific classification, thereby contributing novel insights to the complex decision-making process in calcaneal fracture management [26-28].

Our results demonstrated that treatment outcomes were not determined solely by the surgical technique but were strongly influenced by fracture morphology as defined by the Essex-Lopresti classification. Type 1 subtypes generally achieved balanced results, though Type 1C fractures yielded lower AOFAS scores, consistent with prior findings that intra-articular extension and joint surface irregularities predispose to poorer long-term function [27, 29]. Type 2A fractures showed the most favorable functional recovery with Steinmann pin fixation, a finding that supports the notion that minimally invasive fixation may preserve joint congruity and minimize soft-tissue complications, as supported by previous studies [30-33]. In contrast, Type 2B fractures were consistently associated with poor functional recovery, regardless of the treatment modality, which aligns with earlier reports emphasizing the detrimental impact of subtalar incongruity and severe comminution [34, 35]. For Type 2C fractures, Steinmann pin fixation and plating produced comparable outcomes, reinforcing evidence that percutaneous methods can be as effective as plating in selected cases while reducing risks of wound complications [31, 32]. Consistent with this, 3D model–assisted percutaneous fixation has been shown to significantly reduce wound complications compared with extensile lateral ORIF, while shortening operative time and hospital stay [30].

Radiological evaluation revealed that Böhler’s angle was highest in the conservatively treated group and lowest in the plating group, though these differences did not significantly correlate with AOFAS scores. Our observations are consistent with previous systematic and clinical research on the management of calcaneal fractures. Earlier evidence indicated that there is no conclusive proof supporting the superiority of operative treatment over conservative management in terms of long-term functional recovery, while also highlighting the persistent risk of wound complications and the limited functional improvement achieved after surgery [22, 23]. In agreement with these findings, the present results suggest that fracture morphology and subtype may be more decisive factors influencing outcomes than the specific fixation technique used. This underscores the limited predictive value of radiographic parameters alone and emphasizes the need for integrating functional and dynamic assessments—such as gait analysis—into outcome evaluation. [15,36]. The high incidence of postoperative wound complications, subtalar arthritis, and persistent pain reported in recent literature further supports the need for treatment strategies that minimize soft-tissue disruption and address long-term biomechanical deficits [19-21]. These findings also highlight that achieving radiographic alignment does not necessarily translate into complete functional recovery, as chronic pain and stiffness may persist despite anatomically satisfactory reduction [19-21]. While Gissane’s angle did not differ significantly between groups, the association between Böhler’s angle and clinical outcome observed in other reports [18,35] suggests that initial fracture severity rather than surgical technique often dictates prognosis.

Dynamic gait analysis added further depth to the interpretation of outcomes, consistent with previous pedobarographic studies using quantitative footscan analysis in postoperative calcaneal fractures [37]. Conservative patients exhibited the highest plantar pressure values, whereas plate fixation patients demonstrated the lowest [10–12]. In particular, prolonged swing and step duration observed in Type 2B and 2C subgroups showed a tendency to persist rather than achieving full recovery, which may contribute to chronic pain and functional limitation in the long term. This pattern could potentially be associated with fear of movement (kinesiophobia), as previously reported following lower extremity fractures [38, 39]. However, this remains a hypothesis that warrants further investigation. These observations highlight the importance of integrating pedographic evaluation into postoperative follow-up, as radiological union alone does not capture the functional burden of calcaneal fractures [13, 40].When compared with large randomized controlled trials and meta-analyses, our findings resonate with the general consensus that neither operative nor nonoperative treatment demonstrates universal superiority in displaced intra-articular calcaneal fractures [29, 34, 35]. Instead, individualized treatment strategies, guided by both fracture subtype and patient-specific factors, appear most appropriate. For instance, Steinmann pin fixation may be preferable for Type 2A fractures with preserved joint morphology, whereas plate fixation may remain necessary for selected highly displaced Type 2C patterns, despite higher complication risks [30-33].

The choice of the Essex-Lopresti classification in our study warrants discussion, particularly in the context of the more contemporary Sanders CT-based system. While we acknowledge that the Sanders classification offers superior granularity for characterizing posterior facet comminution and is a proven prognostic tool [2, 4], our use of the Essex-Lopresti system was deliberate. It provided a consistent, radiographic framework for our retrospective cohort and, crucially, categorizes fractures by the primary fracture line and mechanism of injury [41]. We hypothesized that these fundamental morphological patterns have a direct bearing on global calcaneal anatomy and hindfoot biomechanics—the primary focus of our gait analysis. The two systems are complementary; Essex-Lopresti joint depression types (2A-2C) often correlate with higher degrees of comminution in Sanders types [42, 43]. Had a CT-based classification been uniformly available, it might have refined our analysis of articular surface restoration. However, our key findings on the differential outcomes of treatments across Essex-Lopresti subtypes suggest that the primary fracture morphology it describes remains a significant and practical predictor of functional recovery.

This study has several limitations that should be acknowledged. First, its retrospective and non-randomized design inherently limits the ability to establish causality between treatment method and outcome. Because treatment allocation was determined by the attending surgeon rather than by randomization, there is potential for selection bias. The choice among conservative management, Steinmann pin fixation, and plate fixation may have been influenced by factors such as fracture morphology, soft-tissue condition, comorbidities, and patient activity level. This confounding by indication could partially account for differences in functional or gait outcomes and should be considered when interpreting the comparative results. Second, the group sizes were unequal, particularly with a predominance of Steinmann pin cases, which may have reduced statistical power. Third, while Essex-Lopresti classification was applied retrospectively, additional systems (e.g., Sanders CT classification) could provide complementary stratification. Finally, long-term follow-up was limited to a minimum of 18 months, yet functional recovery could potentially continue to evolve beyond 24 months, although this was not evaluated in the present study.

## Conclusion

This study demonstrates that the Essex–Lopresti fracture subtype plays a decisive role in clinical, radiographic, and gait outcomes after calcaneal fractures. Type 2A fractures achieved the best results with Steinmann pin fixation, supporting the efficacy of minimally invasive approaches in selected cases, whereas Type 2B fractures consistently showed poor recovery regardless of treatment. Radiographic parameters such as Böhler’s and Gissane’s angles did not reliably predict functional outcomes, highlighting the need to complement radiologic assessment with gait analysis. Overall, these findings support a subtype-specific, function-oriented approach to optimize management and rehabilitation strategies for intra-articular calcaneal fractures.

## Electronic Supplementary Material

Below is the link to the electronic supplementary material.

Supplementary Material 1

## Data Availability

The datasets generated and/or analyzed during the current study are available from the corresponding author upon reasonable request.
