# Supplementary material for: Comparative outcomes of conservative, steinmann pin, and plate fixation in calcaneal fractures: a subtype-based evaluation according to the essex-lopresti classification
Source: J Orthop Surg Res. 2025 Dec 2;20:1090. doi: 10.1186/s13018-025-06533-1 (PMC12746641; doi:10.1186/s13018-025-06533-1)
Supplement: Supplementary file 1 — Supplementary Material 1 [file 13018_2025_6533_MOESM1_ESM.docx]

**Supplementary File S1**

**Institutional Treatment Protocols for Calcaneal Fracture Management**

**Overview**

The following treatment protocols reflect the standardized institutional guidelines applied at our tertiary university hospital for the management of intra-articular calcaneal fractures. These protocols were followed consistently across all patients included in the present study, with adjustments tailored to the fracture subtype, soft-tissue status, and selected intervention (conservative treatment, Steinmann pin fixation, or plate fixation).

**1. Conservative Treatment Protocol**

**1.1. Initial Management**

- Rest, ice, compression, elevation

- Analgesia and anti-edema management as required

- Short-leg posterior splint applied for initial swelling control

**1.2. Immobilization**

- Transition to below-knee cast within 3–5 days

- Duration: 6–8 weeks of non–weight-bearing immobilization

- Cast integrity and skin condition assessed at 2-week intervals

**1.3. Weight-Bearing Progression**

- Weeks 0–6/8: Strict non–weight-bearing

- Weeks 8–10: Partial weight-bearing using crutches

- Weeks 10–12: Full weight-bearing as tolerated

**1.4. Rehabilitation**

- Passive/active ankle range of motion (ROM) exercises after cast removal

- Strengthening program

- Gait retraining when full weight-bearing is tolerated

- Full activity at 4–6 months as recovery permits

**2. Steinmann Pin Fixation Protocol**

**2.1. Surgical Indications**

- Posterior facet displacement amenable to closed reduction

- Suitable soft-tissue envelope

- Essex-Lopresti Types 1A–2C

**2.2. Surgical Procedure**

- Closed reduction under fluoroscopy

- Percutaneous insertion of Steinmann pins

- Pins maintained for 6–8 weeks

**2.3. Postoperative Immobilization**

- Short-leg cast or boot postoperatively

- Non–weight-bearing for 6–8 weeks

**2.4. Weight-Bearing Progression**

- Weeks 0–8: Non–weight-bearing

- Weeks 8–10: Partial weight-bearing

- Weeks 10–12: Full weight-bearing

**2.5. Rehabilitation**

- ROM after cast/pin removal

- Strengthening program

- Balance training from week 10 onward

**3. Plate Fixation Protocol**

**3.1. Surgical Indications**

- Displaced intra-articular fractures unsuitable for closed reduction

- Severe comminution or loss of calcaneal height

- Essex-Lopresti Types 2A–2C

**3.2. Surgical Procedure**

- Sinus tarsi or extensile lateral approach

- Restoration of height, width, and joint congruity

- Fixation with anatomical plate and screws

**3.3. Immobilization**

- Splint for 10–14 days

- Transition to removable boot

- Non–weight-bearing for 6–8 weeks

**3.4. Weight-Bearing Progression**

- Weeks 0–6/8: Non–weight-bearing

- Weeks 8–10: Partial weight-bearing

- Weeks 10–12+: Progressive full weight-bearing

**3.5. Rehabilitation**

- ROM at 2–3 weeks post-op

- Strengthening at 6 weeks

- Gait training with partial weight-bearing

**4. General Postoperative Care (All Treatment Groups)**

**4.1. Follow-Up Schedule**

- 2 weeks: Wound/cast inspection

- 6–8 weeks: Radiographic assessment

- 3, 6, 12 months: Functional and gait evaluation

**4.2. Complication Monitoring**

- Soft-tissue compromise

- Pin site infection

- Subtalar stiffness

- Chronic pain/gait asymmetry

- Radiographic loss of reduction

**5. Rehabilitation Principles**

- Subtalar ROM focus

- Strengthening of intrinsic and extrinsic foot muscles

- Balance training

- Gradual return to work/sport
